# Correspondence between the Energy Equipartition Theorem in Classical Mechanics and Its Phase-Space Formulation in Quantum Mechanics

**DOI:** 10.3390/e25060939

**Published:** 2023-06-15

**Authors:** Esteban Marulanda, Alejandro Restrepo, Johans Restrepo

**Affiliations:** 1 Instituto de Física, Universidad de Antioquia, Calle 70 No. 52-21, Medellín 050010, Colombia; 2 Group of Magnetism and Simulation, Instituto de Física, Universidad de Antioquia, A.A. 1226, Medellín 050010, Colombia

**Keywords:** energy equipartition theorem, phase-space quantum mechanics, statistical mechanics

## Abstract

In classical physics, there is a well-known theorem in which it is established that the energy per degree of freedom is the same. However, in quantum mechanics, due to the non-commutativity of some pairs of observables and the possibility of having non-Markovian dynamics, the energy is not equally distributed. We propose a correspondence between what is known as the classical energy equipartition theorem and its counterpart in the phase-space formulation in quantum mechanics based on the Wigner representation. Further, we show that in the high-temperature regime, the classical result is recovered.

## 1. Introduction

The energy equipartition theorem is one of the most important results in the classical theory of statistical mechanics due to its quantitative predictions and applicability in many areas of physics [1]. However, few references exist for an extension of it when quantum effects become relevant, that is, mainly with low-temperature phenomena [2]. A better understanding of the energetic distributions in this regime is then necessary for progressing in theoretical aspects and applications with quantum systems.

Currently, the authors working in the area agree that the equipartition of energy no longer holds in the quantum regime, and the energetic distribution follows a better-called energy partition theorem supported in the construction of a distribution function [3,4,5]. Applications to a few models have been made with satisfactory results and holding the correspondence with the classical theorem at a high-temperature regime [4,6,7]. Nonetheless, these works are based on the same conceptual and mathematical framework, and none of them is formulated in phase-space formalism. Therefore, in order to create a true correspondence between the energy equipartition theorem in classical mechanics, it is necessary to reformulate the quantum version in phase space.

In this article a novel approach is implemented; namely, we derive the partition theorem in the phase space of quantum mechanics through the Wigner representation. First, a review and certain mathematical manipulations are made to the classical statement of the theorem so that with the aid of some results in the phase-space formulation, the version of the theorem is shown straightforwardly in a completely analog manner. The results derived are tested and validated, applying them to the harmonic oscillator in both high and low-temperature regimes for the particular case of a weak coupling limit.

## 2. Classical Version of the Theorem

Consider a system composed of *N* particles in thermal equilibrium and described by the set of generalized coordinates {$q_{i}$, $p_{i}$} where i=1,2,…,fN with *f* being the number of degrees of freedom per particle. The classical Hamiltonian of the system is $H_{S}\left(q,p\right)$ where *q* and *p* represent all the generalized coordinates, so the 3D energy equipartition theorem reads as follows [1]

(1) $$\langle q_{i}\frac{\partial H_{S}}{\partial q_{i}}\rangle =\frac{1}{h^{3N}}\int d^{N}q\;d^{N}p\;\rho \left(q,p\right)\;q_{i}\frac{\partial H_{S}}{\partial q_{i}}=\frac{1}{\beta },$$

(2) $$\langle p_{i}\frac{\partial H_{S}}{\partial p_{i}}\rangle =\frac{1}{h^{3N}}\int d^{N}q\;d^{N}p\;\rho \left(q,p\right)\;p_{i}\frac{\partial H_{S}}{\partial p_{i}}=\frac{1}{\beta },$$

where *h* is the Planck constant, $\beta ={\left(k_{B}T\right)}^{-1}$ where $k_{B}$ is the Boltzmann constant, q and p denote all the coordinates per particle, and ρ(q,p) is the corresponding phase-space density in any of the Gibbs ensembles [1]. Notice that the coordinates $q_{i}$ and $p_{i}$ have not been merged into a single coordinate $x_{i}$ as is customary in the demonstration of this theorem; this has a purpose, as will be shortly shown. In the particular case in which the density distribution is given by the canonical ensemble, $\rho \left(q,p\right)=\exp(-\beta H_{S}\left(q,p\right))/Z$ where the partition function is explicitly $Z=\frac{1}{h^{3N}}\int d^{N}q\;d^{N}p\;\exp-\left(\beta H_{S}\left(q,p\right)\right)$, it is possible to conceive of an alternative version of this theorem for the case in which β does not correspond with the known expression, but with a modified function of temperature $\beta_{mod}=\beta_{mod}\left(T\right)$. Then, Equations (1) and (2) read as follows

(3) $$\langle q_{i}\frac{\partial H_{S}}{\partial q_{i}}\rangle =\frac{1}{\beta_{mod}},$$

(4) $$\langle p_{i}\frac{\partial H_{S}}{\partial p_{i}}\rangle =\frac{1}{\beta_{mod}},$$

where now the density distribution is given by $\rho_{mod}\left(q,p\right)=\exp\left(-\beta_{mod}H_{S}\left(q,p\right)\right)/Z_{mod}$.

Let now assume that the Hamiltonian of the system can be separated into two functions according to,

(5) $$H_{S}\left(q,p\right)=F\left(q\right)+G\left(p\right),$$

so we will be able to transform Equations (1) and (2) into more favorable forms for the connection with the quantum mechanical phase-space formulation. Let us now consider the following Hamilton equations

(6) $${\dot{q}}_{i}=\frac{\partial H_{S}}{\partial p_{i}}={\left\{q_{i},H_{S}\right\}}_{PB},$$

(7) $${\dot{p}}_{i}=-\frac{\partial H_{S}}{\partial q_{i}}={\left\{p_{i},H_{S}\right\}}_{PB},$$

where ${\left\{,\right\}}_{PB}$ is the Poisson bracket defined for quantities A(q,p) and B(q,p) as

(8) $${\left\{A,B\right\}}_{PB}=\sum_{i}\left(\frac{\partial A}{\partial q_{i}}\frac{\partial B}{\partial p_{i}}-\frac{\partial A}{\partial p_{i}}\frac{\partial B}{\partial q_{i}}\right),$$

including a third function C(q,p), the following identity can be demonstrated

(9) $${\left\{AB,C\right\}}_{PB}=A{\left\{B,C\right\}}_{PB}+{\left\{A,C\right\}}_{PB}B,$$

applying these results for the quantities $q_{i}\frac{\partial H_{S}}{\partial q_{i}}$ and $p_{i}\frac{\partial H_{S}}{\partial p_{i}}$, using Equations (5)–(7) and identity (9), it can be shown that

(10) $$q_{i}\frac{\partial H_{S}}{\partial q_{i}}=-q_{i}{\left\{p_{i},H_{S}\right\}}_{PB}=-q_{i}{\left\{p_{i},F\left(q\right)\right\}}_{PB}=-{\left\{q_{i}p_{i},F\left(q\right)\right\}}_{PB},$$

(11) $$p_{i}\frac{\partial H_{S}}{\partial p_{i}}=p_{i}{\left\{q_{i},H_{S}\right\}}_{PB}=p_{i}{\left\{q_{i},G\left(p\right)\right\}}_{PB}={\left\{p_{i}q_{i},G\left(p\right)\right\}}_{PB}.$$

Replacing Equations (10) and (11) in (3) and (4), respectively, one obtains

(12) $$\langle q_{i}\frac{\partial H_{S}}{\partial q_{i}}\rangle =-\int d^{N}q\;d^{N}p\;\frac{\exp(-\beta_{mod}H_{S}\left(q,p\right))}{Z_{mod}}\;{\left\{q_{i}p_{i},F\left(q\right)\right\}}_{PB}=\frac{1}{\beta_{mod}},$$

(13) $$\langle p_{i}\frac{\partial H_{S}}{\partial p_{i}}\rangle =\int d^{N}q\;d^{N}p\;\frac{\exp(-\beta_{mod}H_{S}\left(q,p\right))}{Z_{mod}}\;{\left\{p_{i}q_{i},G\left(p\right)\right\}}_{PB}=\frac{1}{\beta_{mod}}.$$

These are the algebraic forms of the modified energy equipartition theorem that will allow us henceforth to easily connect with the quantum mechanical phase-space formulation. In the case when $\beta_{mod}=\beta$, we recover the usual equipartition theorem.

## 3. Density Operator in Quantum Mechanics

For a non-factorizing initial state, it is known that the total state of a system (S) and an environment (E) in quantum mechanics can be described by the thermal equilibrium state through the density operator [8]

(14) $$\hat{\rho }=\exp(-\beta \hat{H})/Z,$$

where $\hat{H}$ is the total Hamiltonian of the system plus environment acting on the total Hilbert space $H=H_{S}⊗H_{E}$ and *Z* the total partition function. Explicitly, the total Hamiltonian is $\hat{H}={\hat{H}}_{S}+{\hat{H}}_{E}+\hat{V}$, where ${\hat{H}}_{S}$, ${\hat{H}}_{E}$ and $\hat{V}$ represent the Hamiltonian of the system, the environment, and the mutual interaction, respectively. The interaction $\hat{V}$ can always be written in a diagonal decomposition of a set of operators $\left\{{\hat{S}}_{\alpha }\right\}$ acting only on the system and a set $\left\{{\hat{B}}_{\alpha }\right\}$ acting only on the environment, such that $\hat{V}=\sum_{\alpha }{\hat{S}}_{\alpha }⊗{\hat{B}}_{\alpha }$ [9]. The description of the system of interest is given by the reduced density matrix; this object contains all the information that can be extracted by an observer of the system [9]. It can be shown that the reduced density matrix at the second order can be written as [10]

(15) $${\hat{\rho }}_{s}\propto e^{-\beta {\hat{H}}_{S}}\left[1+\frac{1}{\hbar }\sum_{\alpha }\int_{0}^{\hbar \beta }d\tau_{1}\int_{0}^{\tau_{1}}d\tau_{2}{\hat{S}}_{\alpha }\left(-i\tau_{1}\right){\hat{S}}_{\alpha }\left(-i\tau_{2}\right)k_{\alpha }\left(\tau_{1}-\tau_{2}\right)\right],$$

where $\hbar k_{\alpha }\left(\tau \right)={\langle {\hat{B}}_{\alpha }\left(-i\tau \right){\hat{B}}_{\alpha }\left(0\right)\rangle }_{B}$ is the two-time correlation function of the environment operators. Equation (15) shows that, in general, it is not possible to describe the influence of the bath in a system using the Gibbs state as in the case of classical mechanics [10]. In order to give a simple illustration in Section 6, we consider the weak coupling limit. The reduced density matrix, in this case, is the Gibbs state given by

(16) $${\hat{\rho }}_{S}=\exp\left(-\beta {\hat{H}}_{S}\right)/Z_{S},$$

with $Z_{S}={\mathrm{Tr}}_{S}\left(\exp\left(-\beta {\hat{H}}_{S}\right)\right)$ being the canonical partition function. On the other hand, it is useful to note that there is a relationship between the propagator $K\left(q_{f},t,q_{i},0\right)=\langle q_{f}\left|\exp\left(-\frac{it}{\hbar }{\hat{H}}_{S}\right)\right|q_{i}\rangle$ formulated in terms of path integrals [11] and the matrix elements in the position basis of Equation (16). Such a relation is evident when we replace t→−iℏβ in the propagator, i.e, $K\left(q_{f},-i\hbar \beta ,q_{i},0\right)=\langle q_{f}\left|\exp\left(-\beta {\hat{H}}_{S}\right)\right|q_{i}\rangle$, then one obtains

(17) $$\langle q_{f}\left|{\hat{\rho }}_{S}\right|q_{i}\rangle =\frac{1}{Z_{S}}\langle q_{f}\left|\exp\left(-\beta {\hat{H}}_{S}\right)\right|q_{i}\rangle =\frac{1}{Z_{S}}\int Dq\;\exp\left(-\frac{1}{\hbar }S^{E}\left[q\right]\right),$$

where the integral is a functional integral running over all the functions satisfying the boundary conditions $q\left(0\right)=q_{i}$, $q\left(\hbar \beta \right)=q_{f}$ and $S^{E}\left[q\right]=\int_{0}^{\hbar \beta }dsH_{S}\left(q\left(s\right),p\left(s\right)\right)$ is the action of the system.

## 4. Phase-Space Formulation of Quantum Mechanics

There are different approaches to the phase-space formulation of quantum mechanics that try to associate a phase-space distribution function, for example, the Glauber–Sudarshan, Kirkwood, Husimi *Q*-representations [12,13], etc. There is a particular approach that consists in associating every quantum observable $\hat{O}\left(\hat{q},\hat{p}\right)$ to a phase-space function $O_{W}\left(q,p\right)$ (also called symbol) by means of a bijection Φ, called the Weyl transformation and defined as [14]

(18) $$\Phi \left(\hat{O}\left(\hat{q},\hat{p}\right)\right)=O_{W}\left(q,p\right)=\int d^{N}u\;\exp\left(-\frac{i}{\hbar }p\cdot u\right)\langle q+\frac{u}{2}|\hat{O}\left(\hat{q},\hat{p}\right)|q-\frac{u}{2}\rangle ,$$

where

(19) $$\Phi (\hat{q})=q,$$

(20) $$\Phi (\hat{p})=p,$$

are the corresponding symbols of position and momentum operators. The Wigner distribution W(q,p) is defined as the Weyl symbol of the density operator $\hat{\rho }$ by

(21) $$W\left(q,p\right)=\rho_{W}\left(q,p\right)=\frac{1}{{\left(2\pi \hbar \right)}^{f}}\int d^{N}u\;\exp\left(-\frac{i}{\hbar }p\cdot u\right)\langle q+\frac{u}{2}|\hat{\rho }\left(\hat{q},\hat{p}\right)|q-\frac{u}{2}\rangle ,$$

is the corresponding phase-space distribution. This allows us to compute averages in a similar way as in classical mechanics by means of

(22) $$\langle \hat{O}\rangle =\int d^{N}q\;d^{N}p\;W\left(q,p\right)O_{W}.$$

Our purpose is to create an analogy between the classical theorem and its counterpart in phase-space quantum mechanics in the particular case where we analyze the energetic distribution of the system. Thus the Hamiltonian that appears in the classical version of the energy equipartition theorem is the Hamiltonian of the system ${\hat{H}}_{S}$. Therefore, it is of interest to calculate the same average in quantum mechanics formulated in phase space in order to create an analogy. To do that, we consider $\hat{q_{i}}=\hat{q_{i}}⊗I$, $\hat{p_{i}}=\hat{p_{i}}⊗I$ and $\frac{\partial {\hat{H}}_{s}}{\partial {\hat{q}}_{i}}=\frac{\partial {\hat{H}}_{s}}{\partial {\hat{q}}_{i}}⊗I$, $\frac{\partial {\hat{H}}_{s}}{\partial {\hat{p}}_{i}}=\frac{\partial {\hat{H}}_{s}}{\partial {\hat{p}}_{i}}⊗I$, so we have

(23) $$\langle \hat{q_{i}}\frac{\partial {\hat{H}}_{S}}{\partial {\hat{q}}_{i}}⊗I\rangle ={\mathrm{Tr}}_{S}\left({\hat{\rho }}_{S}\hat{q_{i}}\frac{\partial {\hat{H}}_{S}}{\partial {\hat{q}}_{i}}\right)=\int d^{N}q\;d^{N}p\;W_{s}\left(q,p\right){\left(\hat{q_{i}}\frac{\partial {\hat{H}}_{S}}{\partial {\hat{q}}_{i}}\right)}_{W},\;$$

(24) $$\langle \hat{p_{i}}\frac{\partial {\hat{H}}_{S}}{\partial {\hat{p}}_{i}}⊗I\rangle ={\mathrm{Tr}}_{S}\left({\hat{\rho }}_{S}\hat{p_{i}}\frac{\partial {\hat{H}}_{S}}{\partial {\hat{p}}_{i}}\right)=\int d^{N}q\;d^{N}p\;W_{s}\left(q,p\right){\left(\hat{p_{i}}\frac{\partial {\hat{H}}_{S}}{\partial {\hat{p}}_{i}}\right)}_{W},\;$$

where $W_{s}\left(q,p\right)$ is the Wigner distribution associated with the reduced density matrix of the system.

A Weyl symbol of particular interest is the one associated with the product of two operators given by [14]

(25) $$\begin{array}{c} \Phi \left(\hat{A}\left(\hat{q},\hat{p}\right)\hat{B}\left(\hat{q},\hat{p}\right)\right)=A_{W}\left(q,p\right){⋆}_{M}B_{W}\left(q,p\right)\\ =A_{W}\left(q,p\right)\exp\left(\frac{i\hbar }{2}\left(\overset{\leftarrow }{\partial_{q}}\vec{\partial_{p}}-\overset{\leftarrow }{\partial_{p}}\vec{\partial_{q}}\right)\right)B_{W}\left(q,p\right), \end{array}$$

where $\overset{\leftarrow }{\partial_{q}}$ denotes the derivative with respect to q acting to the left and similarly for the other derivatives. The ${⋆}_{M}$ is called the Moyal product and allows one to define the associated symbol for the commutator ${\left\{,\right\}}_{M}$ known as the Moyal bracket

(26) $$\begin{array}{c} \Phi \left(\left[\hat{A}\left(\hat{q},\hat{p}\right),\hat{B}\left(\hat{q},\hat{p}\right)\right]\right)={\left\{A_{W}\left(q,p\right),B_{W}\left(q,p\right)\right\}}_{M}\\ =\frac{2}{\hbar }A_{W}\left(q,p\right)\sin\left(\frac{\hbar }{2}\left(\overset{\leftarrow }{\partial_{q}}\vec{\partial_{p}}-\overset{\leftarrow }{\partial_{p}}\vec{\partial_{q}}\right)\right)B_{W}\left(q,p\right), \end{array}$$

which, expanded, is expressed as

(27) $$\begin{array}{c} {\left\{A_{W}\left(q,p\right),B_{W}\left(q,p\right)\right\}}_{M}=2\sum_{s=0}^{\infty }\frac{{\left(-1\right)}^{s}}{(2s+1)!}{\left(\frac{-\hbar }{2}\right)}^{2s}\sum_{t=0}^{2s+1}\frac{{\left(-1\right)}^{t}\left(2s+1\right)!}{(2s+1-t)!t!}\\ \times \left[\frac{\partial^{t}}{\partial q^{t}}\frac{\partial^{2s+1-t}A_{W}}{\partial p^{2s+1-t}}\right]\left[\frac{\partial^{2s+1-t}}{\partial q^{2s+1-t}}\frac{\partial^{t}B_{W}}{\partial p^{t}}\right]\\ ={\left\{A_{W}\left(q,p\right),B_{W}\left(q,p\right)\right\}}_{PB}+O\left(\hbar^{2}\right). \end{array}$$

## 5. Version of the Theorem in Phase-Space Formulation of Quantum Mechanics

Proceeding in a similar way as in Section 1, by assuming

(28) $${\hat{H}}_{S}\left(\hat{q},\hat{p}\right)=\hat{F}\left(\hat{q}\right)+\hat{G}\left(\hat{p}\right),$$

and considering the commutator for operators $\hat{A}\left(\hat{q},\hat{p}\right)$ and $\hat{B}\left(\hat{q},\hat{p}\right)$ defined as

(29) $$\left[\hat{A},\hat{B}\right]=\hat{A}\hat{B}-\hat{B}\hat{A},$$

the following identities can be demonstrated [15]

(30) $$\left[\hat{A}\hat{B},\hat{C}\right]=\hat{A}\left[\hat{B},\hat{C}\right]+\left[\hat{A},\hat{C}\right]\hat{B},$$

(31) $$\left[{\hat{q}}_{i},G\left(\hat{p}\right)\right]=i\hbar \frac{dG(\hat{p})}{d{\hat{p}}_{i}},$$

(32) $$\left[{\hat{p}}_{i},F\left(\hat{q}\right)\right]=-i\hbar \frac{dF(\hat{q})}{d{\hat{q}}_{i}},$$

where F and G are arbitrary functions of operators $\hat{q}$ and $\hat{p}$. Thus, using Equations (31) and (32), the quantum mechanical analog of the Hamilton equations can be stated as

(33) $$\frac{d{\hat{q}}_{i}}{dt}=\frac{\partial {\hat{H}}_{S}}{d{\hat{p}}_{i}}=\frac{1}{i\hbar }\left[{\hat{q}}_{i},{\hat{H}}_{S}\right],$$

(34) $$\frac{d{\hat{p}}_{i}}{dt}=-\frac{\partial {\hat{H}}_{S}}{d{\hat{q}}_{i}}=\frac{1}{i\hbar }\left[{\hat{p}}_{i},{\hat{H}}_{S}\right].$$

Similarly, one arrives at

(35) $${\hat{q}}_{i}\frac{\partial {\hat{H}}_{S}}{\partial {\hat{q}}_{i}}=-\frac{{\hat{q}}_{i}}{i\hbar }\left[{\hat{p}}_{i},{\hat{H}}_{S}\right]=-\frac{{\hat{q}}_{i}}{i\hbar }\left[{\hat{p}}_{i},\hat{F}\left(\hat{q}\right)\right]=-\frac{1}{i\hbar }\left[{\hat{q}}_{i}{\hat{p}}_{i},\hat{F}\left(\hat{q}\right)\right],$$

(36) $${\hat{p}}_{i}\frac{\partial {\hat{H}}_{S}}{\partial {\hat{p}}_{i}}=\frac{{\hat{p}}_{i}}{i\hbar }\left[{\hat{q}}_{i},{\hat{H}}_{S}\right]=\frac{{\hat{p}}_{i}}{i\hbar }\left[{\hat{q}}_{i},\hat{G}\left(\hat{p}\right)\right]=\frac{1}{i\hbar }\left[{\hat{p}}_{i}{\hat{q}}_{i},\hat{G}\left(\hat{p}\right)\right].$$

Using substitution in Equations (23) and (24) the result is

(37) $$\begin{array}{c} \langle \hat{q_{i}}\frac{\partial {\hat{H}}_{S}}{\partial {\hat{q}}_{i}}⊗I\rangle =-\int d^{N}q\;d^{N}p\;W_{s}\left(q,p\right){\left(\frac{1}{i\hbar }\left[{\hat{q}}_{i}{\hat{p}}_{i},\hat{F}\left(\hat{q}\right)\right]\right)}_{W}\\ =-\int d^{N}q\;d^{N}p\;W_{s}\left(q,p\right){\left\{q_{i}p_{i},F\left(q\right)\right\}}_{M}, \end{array}$$

(38) $$\begin{array}{c} \langle \hat{p_{i}}\frac{\partial {\hat{H}}_{S}}{\partial {\hat{p}}_{i}}⊗I\rangle =\int d^{N}q\;d^{N}p\;W_{s}\left(q,p\right){\left(\frac{1}{i\hbar }\left[{\hat{p}}_{i}{\hat{q}}_{i},\hat{G}\left(\hat{p}\right)\right]\right)}_{W}\\ =\int d^{N}q\;d^{N}p\;W_{s}\left(q,p\right){\left\{p_{i}q_{i},G\left(p\right)\right\}}_{M}. \end{array}$$

Expanding the Moyal bracket as in (27) and noticing that the $O(\hbar^{2})$ vanishes since derivatives of third-order or higher cancel out, one arrives at

(39) $$\begin{array}{c} \langle \hat{q_{i}}\frac{\partial {\hat{H}}_{S}}{\partial {\hat{q}}_{i}}⊗I\rangle =-\int d^{N}q\;d^{N}p\;W_{s}\left(q,p\right){\left\{q_{i}p_{i},F\left(q\right)\right\}}_{M}\\ =-\int d^{N}q\;d^{N}p\;W_{s}\left(q,p\right){\left\{q_{i}p_{i},F\left(q\right)\right\}}_{PB}, \end{array}$$

(40) $$\begin{array}{c} \langle \hat{p_{i}}\frac{\partial {\hat{H}}_{S}}{\partial {\hat{p}}_{i}}⊗I\rangle =\int d^{N}q\;d^{N}p\;W_{s}\left(q,p\right){\left\{p_{i}q_{i},G\left(p\right)\right\}}_{M}\\ =\int d^{N}q\;d^{N}p\;W_{s}\left(q,p\right){\left\{p_{i}q_{i},G\left(p\right)\right\}}_{PB}. \end{array}$$

Notice the explicit resemblance with the classical version as shown in Equations (1) and (2). The exact same result can be obtained for the environment but with the Wigner distribution function associated with the reduced density matrix of the bath. Equations (39) and (40) are very useful for understanding why in quantum mechanics, generally, there is no energy equipartition per degree of freedom in a system. The key factor is that the Weyl transform of the reduced density matrix does not coincide with the Gibbs state. Moreover, note that, according to Equation (14), the Wigner distribution can be divided into two terms

(41) $$\begin{array}{c} \begin{array}{cc} \; & W_{s}\left(q,p\right)=\int d^{N}u\;\exp\left(-\frac{i}{\hbar }p\cdot u\right)\langle q+\frac{u}{2}|\frac{\exp\left(-\beta {\hat{H}}_{S}\right)}{Z}|q-\frac{u}{2}\rangle \\ +\int d^{N}u\; & \exp\left(-\frac{i}{\hbar }p\cdot u\right)\langle q+\frac{u}{2}|\frac{1}{Z\hbar }\sum_{\alpha }\int_{0}^{\hbar \beta }d\tau_{1}\int_{0}^{\tau_{1}}d\tau_{2}{\hat{S}}_{\alpha }\left(-i\tau_{1}\right){\hat{S}}_{\alpha }\left(-i\tau_{2}\right)k_{\alpha }\left(\tau_{1}-\tau_{2}\right)|q-\frac{u}{2}\rangle . \end{array} \end{array}$$

The first and second terms contain information about the system in a thermal state and the bath correlation, respectively. Then, because of the non-commutativity of the observables in the system, the first term does not coincide with the Gibbs state due to quantum corrections, and the second, in general, at finite temperatures does not vanish even for simple models of bath-system coupling (Legget–Caldeira type) [10]. As we will show in Section 7, these Equations (39) and (40) constitute the energy equipartition theorem only for the special case of the high-temperature regime.

## 6. Application to the Quantum Harmonic Oscillator

As an illustrative example of the proposed method in the article for calculating the energy, let us consider the system to be a harmonic oscillator in the high and low-temperature regimes. First, we calculate the Wigner distribution associated with the reduced density matrix in the weak coupling limit

(42) $$W_{s}\left(q,p\right)=\frac{1}{Z_{S}}\frac{1}{2\pi \hbar }\int du\;\exp\left(-\frac{i}{\hbar }p\cdot u\right)\langle q+\frac{u}{2}\left|\exp\left(-\beta {\hat{H}}_{S}\right)\right|q-\frac{u}{2}\rangle ,$$

with ${\hat{H}}_{S}=\frac{1}{2m}{\hat{p}}^{2}+\frac{1}{2}m\omega^{2}{\hat{q}}^{2}$. As we said in Section 2, the matrix elements of the density operator involved in the integral can be calculated in the imaginary time path integral formalism, i.e., we use the expression of the propagator of a harmonic oscillator, and we replace t=−iℏβ. We do this in the expression provided by [11] to get $\langle q+\frac{u}{2}|\exp\left(-\beta {\hat{H}}_{S}\right)|q-\frac{u}{2}\rangle =\sqrt{\frac{m\omega }{2\pi \hbar \sin h(\hbar \beta \omega )}}\exp(-\frac{m\omega }{\hbar }q^{2}\tan h\left(\frac{\hbar \omega \beta }{2}\right)-\frac{m\omega }{4\hbar }u^{2}\cot h\left(\frac{\hbar \omega \beta }{2}\right))$ and $Z_{S}={\left(2\sin h\left(\frac{\hbar \omega \beta }{2}\right)\right)}^{-1}$. Finally, we replace this in Equation (42) and get

(43) $$\begin{array}{c} W_{s}\left(q,p\right)=\frac{1}{\pi \hbar }\tanh\left(\frac{\hbar \omega \beta }{2}\right)\exp\left(-\frac{\tanh(\frac{\hbar \omega \beta }{2})}{\omega \hbar }\left(\frac{p^{2}}{m}+m\omega^{2}q^{2}\right)\right). \end{array}$$

In the high-temperature regime, we find that the Wigner distribution function is just the reduced density matrix of the system, i.e., the classical phase-space density in the canonical ensemble of a harmonic oscillator,

(44) $$\begin{array}{c} W_{s}\left(q,p\right)=\frac{1}{Z}\exp\left(-\beta \left(\frac{p^{2}}{2m}+\frac{m\omega^{2}}{2}q^{2}\right)\right)=\frac{\exp\left(-\beta H_{S}\left(q,p\right)\right)}{Z}, \end{array}$$

where $Z=\frac{2\pi }{\beta \omega }$. Finally, because Equation (44) corresponds exactly with the probability density function for the canonical ensemble as in the case of classical mechanics, Equations (39) and (40) are equal to $k_{B}T$ using Equations (12) and (13). In the low-temperature regime,

(45) $$\begin{array}{c} \langle \hat{q_{i}}\frac{\partial {\hat{H}}_{S}}{\partial {\hat{q}}_{i}}⊗I\rangle =\lim_{\beta \to \infty }\left(-1\right)\int dq\;dp\;W_{s}\left(q,p\right){\left\{q_{i}p_{i},F\left(q\right)\right\}}_{M}\\ =-\int dq\;dp\;\frac{1}{\pi \hbar }\exp\left(-\frac{2H_{S}\left(q,p\right)}{\omega \hbar }\right){\left\{q_{i}p_{i},F\left(q\right)\right\}}_{PB}=\frac{\hbar \omega }{2}, \end{array}$$

(46) $$\begin{array}{c} \langle \hat{p_{i}}\frac{\partial {\hat{H}}_{S}}{\partial {\hat{p}}_{i}}⊗I\rangle =\lim_{\beta \to \infty }\int dq\;dp\;W_{s}\left(q,p\right){\left\{p_{i}q_{i},G\left(p\right)\right\}}_{M}\\ =\int dq\;dp\;\frac{1}{\pi \hbar }\exp\left(-\frac{2H_{S}\left(q,p\right)}{\omega \hbar }\right){\left\{p_{i}q_{i},G\left(p\right)\right\}}_{PB}=\frac{\hbar \omega }{2}, \end{array}$$

in the last equalities we used Equation (13) where $Z_{mod}=\pi \hbar$, and $\beta_{mod}=\frac{2}{\omega \hbar }$.

This simple example shows that, for this particular case, if one expects to create a true correspondence between the classical equipartition theorem discussed in Section 1 and the theorem in quantum mechanics phase-space formulation, it must be done in the high-temperature regime because, in the low-temperature regime, one gets the modified density distribution. Furthermore, for a model whose coordinates are bilinearly coupled as in [16], explicit expressions are found for Equations (23) and (24) in both regimes for the weak coupling limit, where it is shown that the energy is not distributed equally for the damped harmonic oscillator. This can also be seen from [17] where the Wigner distribution function for Equations (40) and (39) does not correspond with the Gibbs distribution.

## 7. Theorem in the Classical Limit

Motivated by this example, we expect that in the classical limit, the Wigner function described by Equation (42) behaves as the density probability function in the canonical ensemble for any system. In the general case of non-factorizing initial conditions in the high-temperature regime equation, (14) takes the form of Gibbs state, and it can be shown [18] that in this limit, the Wigner distribution function coincides with the same distribution as in the classical case,

(47) $$W_{s}\left(q,p\right)=\frac{\exp(-\beta H_{S}\left(q,p\right))}{Z},$$

where, in this case, we have used the fact that the Wigner distribution function must be normalized. Using this in Equations (39) and (40), we have

(48) $$\begin{array}{c} \langle \hat{q_{i}}\frac{\partial {\hat{H}}_{S}}{\partial {\hat{q}}_{i}}⊗I\rangle \approx -\int d^{N}q\;d^{N}p\;\frac{\exp(-\beta H_{S}\left(q,p\right))}{Z}{\left\{q_{i}p_{i},F\left(q\right)\right\}}_{PB}, \end{array}$$

(49) $$\begin{array}{c} \langle \hat{p_{i}}\frac{\partial {\hat{H}}_{S}}{\partial {\hat{p}}_{i}}⊗I\rangle \approx \int d^{N}q\;d^{N}p\;\frac{\exp(-\beta H_{S}\left(q,p\right))}{Z}{\left\{p_{i}q_{i},G\left(p\right)\right\}}_{PB}. \end{array}$$

This is according to the classical theorem equal to $k_{B}T$ (see Equations (12) and (13)). Then

(50) $$\langle \hat{q_{i}}\frac{\partial {\hat{H}}_{S}}{\partial {\hat{q}}_{i}}⊗I\rangle \approx k_{B}T$$

(51) $$\langle \hat{p_{i}}\frac{\partial {\hat{H}}_{S}}{\partial {\hat{p}}_{i}}⊗I\rangle \approx k_{B}T.$$

These equations represent what we expect to get in the classical limit, i.e., the equipartition theorem. It must be stressed that this result has some approximations, restrictions, and limitations. First, in general, the total state given by Equation (14) must contain some additional conditions resulting from experimentally achievable preparations [19], second the result is derived based on an approximation method derived in [18], and we approximate the product ℏβ tends to zero to guarantee that the state is described by the Gibbs state. The latter is guaranteed formally when T→∞ and as long as the second-order perturbation theory is valid [10]. In this case, no matter what kind of interaction is described by the system and the environment, we get (50) and (51). On the other hand, the result is only valid when one works with separable Hamiltonians, which is a particular case of more general Hamiltonians presented in the literature [20].

## 8. Conclusions

We show that for a quantum system whose Hamiltonian can be separated in a sum of two terms, one dependent exclusively on position coordinates and the other exclusively on momentum, it is possible to establish a correspondence with classical energy equipartition theorem when the quantum mechanical phase space formulation is implemented. In the quantum case, the expression of what would be the equipartition theorem depends on the Wigner distribution of the reduced density matrix instead of the classical phase space density. This expression allows us to understand why in quantum mechanics, in general, there is no energy equipartition theorem: the Wigner distribution function does not coincide with the Gibbs state as would happen in the classical case. The main advantage of our expression, as shown for a quantum harmonic oscillator, is that it allows studying the energetic distribution completely in terms of the Wigner distribution function. Finally, as a limit case, we investigate the high-temperature regime, and, as expected, we recover the classical result.

## Data Availability

Data sharing is not applicable.
